# Genome-wide identification, phylogeny and expressional profile of the Dmrt gene family in Chinese sturgeon (*Acipenser sinensis*)

**DOI:** 10.1038/s41598-024-54899-9

**Published:** 2024-02-20

**Authors:** Yacheng Hu, Ruihua Tan, Xin Zhu, Binzhong Wang, Jingshu Wang, Baifu Guo, Yuan Li, Hejun Du, Yuanjin Yang

**Affiliations:** 1 Chinese Sturgeon Research Institute, China Three Gorges Corporation, Yichang, 443100 Hubei China; 2 Hubei Key Laboratory of Three Gorges Project for Conservation of Fishes, Yichang, 443100 Hubei China; 3 Shanghai Ocean University, Shanghai, 201306 China

**Keywords:** Chinese sturgeon, Dmrt gene, Genomic structure, Gene expression, Gene expression, Genetic linkage study

## Abstract

Chinese sturgeon Dmrt gene family was identified and characterized for the first time. A total of 5 putative Dmrt genes were identified. The gene structure, conserved protein domain and the phylogenetic relationship of Dmrt gene family were systematically analyzed. The expressed profile of Chinese sturgeon Dmrt genes in gonad, pituitary and hypothalamus in the male and female were investigated. The results indicated that the accumulation of Dmrt genes was involved in different tissues, and the expression profile also differed among each Dmrt genes. ASDmrt1A, ASDmrt2, ASDmrt3, and ASDmrtA1 were highly expressed in the testis in comparison with other tissue. This result showed that ASDmrt1A, ASDmrt2, ASDmrt3, and ASDmrtA1 played an important role in the development of testicle, and may be useful tool in distinguishing between male and female of Chinese sturgeon. Our study will provide a basis for additional analyses of Chinese sturgeon Dmrt genes. This systematic analysis provided a foundation for further functional characterization of Dmrt genes with an aim of study of Chinese sturgeon Dmrt gene family.

## Introduction

Chinese sturgeon (*Acipenser sinensis*) is one of the most primitive vertebrates leading to a vital evolutionary position. Chinese sturgeon was once an important commercial fish widely distributed in the Yangtze River and in the China seas^1^. The natural population has declined severely due to habitat degradation caused by anthropogenic activities such as pollution, shipping and over-fishing^2^. According to International Union for the Conservation of Nature (IUCN 2010) data, this species was occurred only rarely in the Yangtze River. Consequently, it is characterized as Critically Endangered in the IUCN and listed under Category I State Protection in China^3^. Lots efforts have been undertaken to support the recovery of Chinese sturgeon, including re-stocking of cultured juveniles, controlled reproduction and establishing a national nature reserve. It is difficult to recover numbers of Chinese sturgeon because of the late sexual maturity (at least 9 and 14 years for males and females, respectively) as well as the reproduction interval of 2–7 years^4^. To save this species from extinction and develop its aquaculture industry for future, artificial propagation has been tried to conduct on the Chinese sturgeon since the later 1980s. The first artificial propagation of cultured Chinese sturgeon was successfully performed in 2009 by the Chinese Sturgeon Research Institute. The success of artificial propagation in Chinese sturgeon is of great significance for protecting this species. However, it is impossible to distinguish females from males morphologically in juvenile individuals. This factor constitutes obstacles not only to the efficacy of artificial propagation but also to the conservation of germplasm resources. Consequently, it is necessary to study the mechanisms of reproduction regulation to resolve this issue.

The double sex and mab-3 related transcription factor (DMRT) family is well-conserved in bilaterian animals^5^. The Dmrt family is characterized by the DM domain which was named from *Drosophila melanogaster* Dsx and *Caenorhabditis elegans* Mab-3 proteins, both of which play important roles in sex differentiation^5^. The existence of Dmrt genes was first formally proposed in the fruit fly, *Drosophila*^6^. Most animals have multiple Dmrt genes. There are eight Dmrt genes (Dmrt1-Dmrt8) in the mammals^7,8^. And Dmrt 7 and Dmrt 8 were reported to be mammalian-specific Dmrt genes^7^. The Dmrt proteins play important roles in various development processes including neurogenesis, somitogenesis, myogenesis and gametogenesis^9–11^. Dmrt 1 is male determination in gonadal somatic cells or a regulator of testicular formation in the vertebrate species^12^. In chickens, the gene dosage of Z-linked dmrt 1 can induce male sex determination^13^. In mice, dmrt 1 is important for somatic-cell masculinization^14^. In female mice, Dmrt1 play roles in oogenesis and Dmrt 4 play roles in folliculogenesis^15,16^. Dmrt 2 participates in somitogenesis and myogenesis in some vertebrates^17,18^. In some bilaterian species, Dmrt3, Dmrt4, Dmrt5, Dmd-5 and Dmrt93B play roles in neurogenesis^19–23^. Only the three types of the Dmrt family genes (*dsx*, *dmrt1* homologs and *mab-3*) are known to function in somatic sex differentiation or sex determination to date. It is still an undecided question as to whether the three types of the Dmrt family genes (*dsx*, *dmrt1* homologs and *mab-3*) are orthologous or not.

The development expression of Dmrt genes in gonad has been reported in several species^24–26^. Dmrt family had been detected in tissues such as the somites (mouse, chick and fish Dmrt2, and chick Dmrt3), nasal placodes (platyfish Dmrt5, Xenopus and platyfish Dmrt4, and mouse and chick Dmrt3) or the central nervous system (Dmrt3, Dmrt4, Dmrt5 and Dmrt6 in chick, Xenopus, mouse and fish). The different profile in the expression of Dmrt genes across species indicate that the function of some members of Dmrt gene and the expression patterns may have shifted during evolution.

In this study, genome-wide investigation of Dmrt genes is performed in Chinese sturgeon. An in silico genome-wide search was used for identifying Chinese sturgeon Dmrt genes. Then, gene structure, conserved protein domains, phylogenetic relationship and expression were systematically analyzed in the putative Chinese sturgeon Dmrt genes to reveal evolutionary and functional features. The results provide useful information for further functional investigations of the Dmrt gene family.

## Materials and methods

### Identification of Chinese sturgeon Dmrt genes

Chinese sturgeon genome was sequenced by our laboratory. All Dmrt-like sequences were identified by tblastn (E = 2e^−5^) against genome sequences, using Dmrt sequences of *Danio rerio* as queries. The identified Dmrt genes were used to back search against the NCBI by blastn to redundant matches. In order to trace the evolutionary origin of the Dmrt genes in teleost fish, Dmrt genes were also identified in other vertebrates from Ensembl (http://www.ensembl.org/) and NCBI databases (http://www.ncbi.nlm.nih.gov), including Zebrafish (*Danio rerio*), Nile Tilapia (*Oreochromis niloticus*), African clawed frog (*Xenopus laevis*), Green Anole (*Anolis carolinensis*), zebra finch (*Taeniopygia guttata*), Chicken (*Gallus gallus*), Mouse (*Mus musculus*), Human (*Homo sapiens*), Amur sturgeon (*Acipenser schrenckii*), Sterlet (*Acipenser ruthenus*), tiger puffer (*Takifugu rubripes*), Bluntsnout bream (*Megalobrama amblycephala*), Chinese softshell (*Pelodiscus sinensis*) and Asian seabass (*Lates calcarifer*).

CDS (Coding Sequence) lengths and number of amino acids of the identified Chinese sturgeon Dmrt genes were obtained from the Chinese sturgeon genome database. The theoretical molecular weight (kDa) and pI (isoelectric points) of each Dmrt protein were calculated using the EXPASY compute pI/MW tool (http://www.expasy.org/tools/). GRAVY (Grand Average of Hydropathy) values were evaluated using the PROTPARAM tool (http://web.expasy.org/protparam/).

### Multiple alignment and phylogenetic analysis of Chinese sturgeon Dmrt genes

The multiple alignment software MEGA 4.0 (http://www.megasoftware.net/mega4/mega.html) was employed to align the amino acid sequences of Dmrt genes from Chinese sturgeon. The full-length amino acid sequences of Dmrt genes derived from Chinese sturgeon, Zebrafish, Nile Tilapia, African clawed frog, Green Anole, zebra finch, Chicken, Mouse, Human, Amur sturgeon, Sterlet, tiger puffer, Bluntsnout bream, Chinese softshell and Asian seabass were used for phylogenetic analysis (Table 1). An unrooted neighbor-joining (NJ) phylogenetic tree was constructed using MEGA4 software with the following parameters: Poisson correction, pairwise deletion, and bootstrap (1000 replicates). The constructed tree file was visualized using figtree software.

**Table 1:** The accession numbers of DMRT used in phylogenetic analysis.

Gene	Accession number	Gene	Accession number
*Danio rerio*-Dmrt1	NP_991191.2	*Mus musculus*-DmrtA1	NP_783578.1
*Danio rerio*-Dmrt2a	NP_571027.1	*Mus musculus*-DmrtA2	AAN10254.1
*Danio rerio*-Dmrt2b	NP_001073445.1	*Mus musculus*-Dmrt6	NP_063925.1
*Danio rerio*-Dmrt3	AAU89440.1	*Mus musculus*-Dmrt7	NP_082008.1
*Danio rerio*-DmrtA2	NP_001007065.2	*Mus musculus*-Dmrt8	NP_081867.1
*Oreochromis niloticus*-Dmrt1	AAF79931.1	*Homo sapiens*-Dmrt1	NP_068770.2
*Oreochromis niloticus*-Dmrt2a	AAN78446.1	*Homo sapiens*-Dmrt2	CAH70589.1
*Oreochromis niloticus*-Dmrt2b	AAX08123.1	*Homo sapiens*-Dmrt3	AAI13585.1
*Oreochromis niloticus*-Dmrt3	XP_003444527.2	*Homo sapiens*-DmrtA1	NP_071443.2
*Oreochromis niloticus*-Dmrt4	AAF79932.2	*Homo sapiens*-DmrtA2	AAI43801.1
*Oreochromis niloticus*-Dmrt5	Q6YHU8.1	*Homo sapiens*-Dmrt6	NP_149056.1
*Oreochromis niloticus*-Dmrt6	XP_003447317.1	*Homo sapiens*-Dmrt7	NP_001035373.1
*Xenopus laevis*-Dmrt1a	NP_001089969.1	*Homo sapiens*-Dmrt8	AAH47596.1
*Xenopus laevis*-Dmrt2	XP_018099312.1	*Acipenser schrenckii*-Dmrt1a	BAZ96609.1
*Xenopus laevis*-Dmrt3	XP_018099314.1	*Acipenser schrenckii*-Dmrt1b	BAZ96610.1
*Xenopus laevis*-Dmrt-4	NP_001084823.1	*Acipenser ruthenus*-Dmrt1	ALL53127.1
*Xenopus laevis*-Dmrt5	AAI70170.1	*Acipenser ruthenus*-Dmrt3	RXM95523.1
*Anolis carolinensis*-Dmrt1	XP_003216601.2	*Takifugu rubripes*-Dmrt1	NP_001033038.1
*Anolis carolinensis*-Dmrt2	XP_003216602.1	*Takifugu rubripes*-DmrtA1	NP_001033037.1
*Anolis carolinensis*-Dmrt3	XP_003216535.1	*Megalobrama amblycephala*-Dmrt1a	AHA85564.1
*Anolis carolinensis*-DmrtA1	XP_001374014.1	*Megalobrama amblycephala*-Dmrt1b	AHA85565.1
*Anolis carolinensis*-Dmrt6	XP_003220348.1	*Megalobrama amblycephala*-Dmrt1c	AHA85566.1
*Taeniopygia guttata*-Dmrt1	XP_002194579.1	*Megalobrama amblycephala*-Dmrt1d	AHA85567.1
*Taeniopygia guttata*-Dmrt2	XP_002194385.1	*Megalobrama amblycephala*-Dmrt3	AJD87235.1
*Taeniopygia guttata*-Dmrt3	XP_002194499.2	*Pelodiscus sinensis*-Dmrt1	AVR54986.1
*Taeniopygia guttata*-Dmrt6	XP_002193808.2	*Pelodiscus sinensis*-DmrtA1	XP_025043468.1
*Gallus gallus*-Dmrt1	ADW41582.1	*Pelodiscus sinensis*-DmrtA2	XP_006126511.2
*Gallus gallus*-Dmrt2	AAZ03502.1	*Pelodiscus sinensis*-Dmrt3	XP_006137927.1
*Gallus gallus*-Dmrt3	XP_429193.2	*Lates calcarifer*-Dmrt2	XP_018523822.1
*Gallus gallus*-Dmrt6	NP_001232910.1	*Lates calcarifer*-Dmrt3	XP_018523823.1
*Mus musculus*-Dmrt1	NP_056641.2	*Lates calcarifer*-DmrtA1	XP_018543487.1
*Mus musculus*-Dmrt2	AAH27669.1	*Lates calcarifer*-DmrtA2	XP_018528863.1
*Mus musculus*-Dmrt3	AAN77230.1		

### Dmrt gene structure construction, protein domain and motif analysis

The MEME online program (http://meme.nbcr.net/meme/intro.html) for protein sequence analysis was used to identify conserved motifs in the identified Chinese sturgeon Dmrt proteins^27^. The optimized parameters were employed as the following: the number of repetitions, any; the maximum number of motifs, 10; and the optimum width of each motif, between 6 and 100 residues. The exon–intron organization of Chinese sturgeon Dmrt genes was determined by comparing predicted coding sequences with their corresponding full-length sequences using the online program Gene Structure Display Serve (GSDS: http://gsds.cbi.pku.edu.cn)^28^.

### The expression profile of ASDmrt genes in hypothalamus, pituitary and gonad of male and female Chinese sturgeon

Details about the transcriptome data derived from hypothalamus, pituitary and gonad of male and female Chinese sturgeon were described in Du et al*.*^29^. 15 Chinese sturgeon individuals were used for analysis the expression profile of ASDmrt genes, including 9 individuals (3 females and 6 males over 4 years old) in stage II and 6 individuals (1 year old) in stage I. In the study of Du et al*.*^29^, the sex cannot be distinguished by a histochemical assay for gonads in stage I, while it can be easily distinguished in stage II in Chinese sturgeon. The transcript abundance of ASDmrt genes was calculated as fragments per kilobase of exon model per million mapped reads (FPKM). The heatmaps were created by HemI1.0 based on the transformed data of log 2 (FPKM + 1) values. The transcriptome data used in this study could also be obtained on the website (https://identifiers.org/ncbi/insdc:GGYF01000000).

Then, we further analyzed the expression patterns of five dmrt family genes by real-time quantitative PCR (qPCR) on five tissues of three female and male 2-year-old Chinese sturgeon individuals, including pituitary, hypothalamus, muscle, gonad, and brain. The gonads of 2-year-old female and male Chinese sturgeon were taken for gonadal histological analysis. The sex identification of Chinese sturgeon according to the identification of sex-linked marker (F: TAAAGGGAGACGGCAGAT; R: CAGGAAAGGCAAGGATGT), which is developed by our laboratory. The sex-linked marker can be amplified on female Chinese sturgeon, but not on male Chinese sturgeon, and is not limited by age. Total RNA of all tissues were extracted using TRIzol reagent according to the manufacturer’s instructions. The First-strand cDNA Synthesis Kit (TaKaRa, Tokyo, Japan) was used for reverse transcription. The primer sequences used in qPCR are listed in Table 2. The Applied Biosystems Quant-Studio™ 5 platform (Thermo Fisher Scientific, Waltham, USA) was used for performing qPCR. The qPCR was performed using in a 20 µl reaction mixture, including 1 µl of cDNA, 1 µl of each primers (Table 2), 10 µl of the SYBR® Green Master Mix (Applied Biosystems, Carlsbad, USA), and ultrapure water. The temperature cycle protocol for amplification was: 50 °C for 2 min for Heated-labile Uracil-DNA Glycosylase (UDG) activation and then 95 °C for 2 min, followed by 40 cycles of 95 °C for 15 s, 60 °C for 30 s and 72 °C for 30 s, followed by dissociation curve analysis to verify the amplification of a single product. Chinese sturgeon β-Actin (Table 2) was used as a positive control for the qPCR analysis to determine the template concentration and to provide an external control for qPCR under the same reaction conditions. Each targeted gene was analyzed in triplicate wells in three Chinese sturgeon individuals (biological replicates). The relative quantification values for the target gene and reference gene were calculated by the standard 2^−ΔΔCt^ method after normalization against the β-Actin gene. The standard error and mean of the measurements (M ± SEM) were calculated from the biological and technological replicates. Statistical significance was measured using the independent samples t-test in SPSS 17.0, with P < 0.05 indicating significance.

**Table 2:** Primers for PCR amplification.

Primers	Sequences(5′–3′)
Dmrt3F	GAAAGCCCTGAAGTGGTCC
Dmrt3R	GATGTTGTTCTCCGTGGTGT
DmrtA1F	GCTGCCATCACCCTCTGT
DmrtA1R	TTTGCTGCGACCACCG
DmrtA2-F	AAGCAGATGAACGCCATAGACAT
DmrtA2-R	TGCCCGTTGTTGTTGAGGAT
Dmrt1A-F	AATCAACTCAGGCGTGCTCT
Dmrt1AR	ATCTGCCACCCTGTTCCAC
Dmrt2F	AAGGCACGAAACCACTCC
Dmrt2R	AAACAGAAAGCGATGACCAG
β-Actin-F	TTATGCCCTGCCCCACGCTATC
β-Actin-R	CGTGTGAAGTGGTAAGTCCGT

### Histology

The female and male 1, 2, 4-year-Chinese sturgeon were used for histological analysis to study early gonadal development. The gonad of female and male 1, 2, 4-year-Chinese sturgeon were taken for fixation in Bouin’s solution. Then, the gonad were trimmed to 3-mm pieces and refixed in Bouin’s solution for 24 h. We used the standard paraffin embedding method to cut those samples into 4-mm thickness, used hematoxylin–eosin (HE) to stain those samples. The digital camera (DP-73, Olympus) and the light microscope (BX-51, Olympus) were used to take images of those samples.

## Result

### Genome-wide analysis of Chinese sturgeon

In this present study, totally 5 Dmrt genes were isolated from the genome of Chinese sturgeon and 4, 6, 5, 5, 4, 8 and 8 Dmrt genes were found in Zebrafish, Nile Tilapia, African clawed frog, Green Anole, zebra finch, Mouse and Human respectively (Table 3). The gene abundance of Dmrt genes in different fish species in compared in Table 3. The identified Dmrt genes in surveyed vertebrates are shown in Table 1.

**Table 3:** Dmrt gene orthologs in the genomes of zebrafish, Nile Tilapia, African clawed frog, Green Anole, zebra finch, Mouse, Human and Chinese sturgeon.

Dmrt genes	Zebrafish	Nile Tilapia	African clawed frog	Green Anole	zebra finch	Mouse	Human	Chinese sturgeon
Dmrt1a	✓	✓	✓	✓	✓	✓	✓	✓
Dmrt2	✓	✓	✓	✓	✓	✓	✓	✓
Dmrt3	✓	✓	✓	✓	✓	✓	✓	✓
Dmrt4(DmrtA1)		✓	✓	✓	–	✓	✓	✓
Dmrt5(DmrtA2)	✓	✓	✓	–	–	✓	✓	✓
Dmrt6 (DmrtB1)	–	✓	–	✓	✓	✓	✓	–
Dmrt7(DmrtC2)	–	–	–	–	–	✓	✓	–
Dmrt8(DmrtC1)	–	–	–	–	–	✓	✓	–

Gene characteristics, including the length of the protein sequence, the putative molecular weights, theoretical isoelectric point and with the grand average of hydropathy values were analyzed (Table 4). Among the 5 ASDmrt proteins, ASDmrt2 was identified to be the largest protein with 505 amino acid (aa), whereas the smallest one was ASDmrt1A (328 aa). The putative molecular weights of the proteins ranged from 35.8817 (ASDmrt1a) to 56.24052 kDa (ASDmrt2), and theoretical isoelectric point ranged from 6.64 (ASDmrt3) to 8.89 (ASDmrtA1). All of the Chinese sturgeon Dmrt genes were hydrophilic, with the grand average of hydropathy values < 0.

**Table 4:** Dmrt family genes in Chinese sturgeon genome and their sequence characteristics.

Gene	Length (aa)	Mol. wt (KDa)	pI	GRAVY
ASDmrt1A	328	35.8817	7.53	− 0.735
ASDmrt2	505	56.24052	8.53	− 0.615
ASDmrt3	452	49.73173	6.64	− 0.617
ASDmrtA1	450	48.65503	8.89	− 0.505
ASDmrtA2	445	47.84484	8.35	− 0.505

### Chinese sturgeon Dmrt genes sequences alignment and phylogenetic

**Figure 1:**
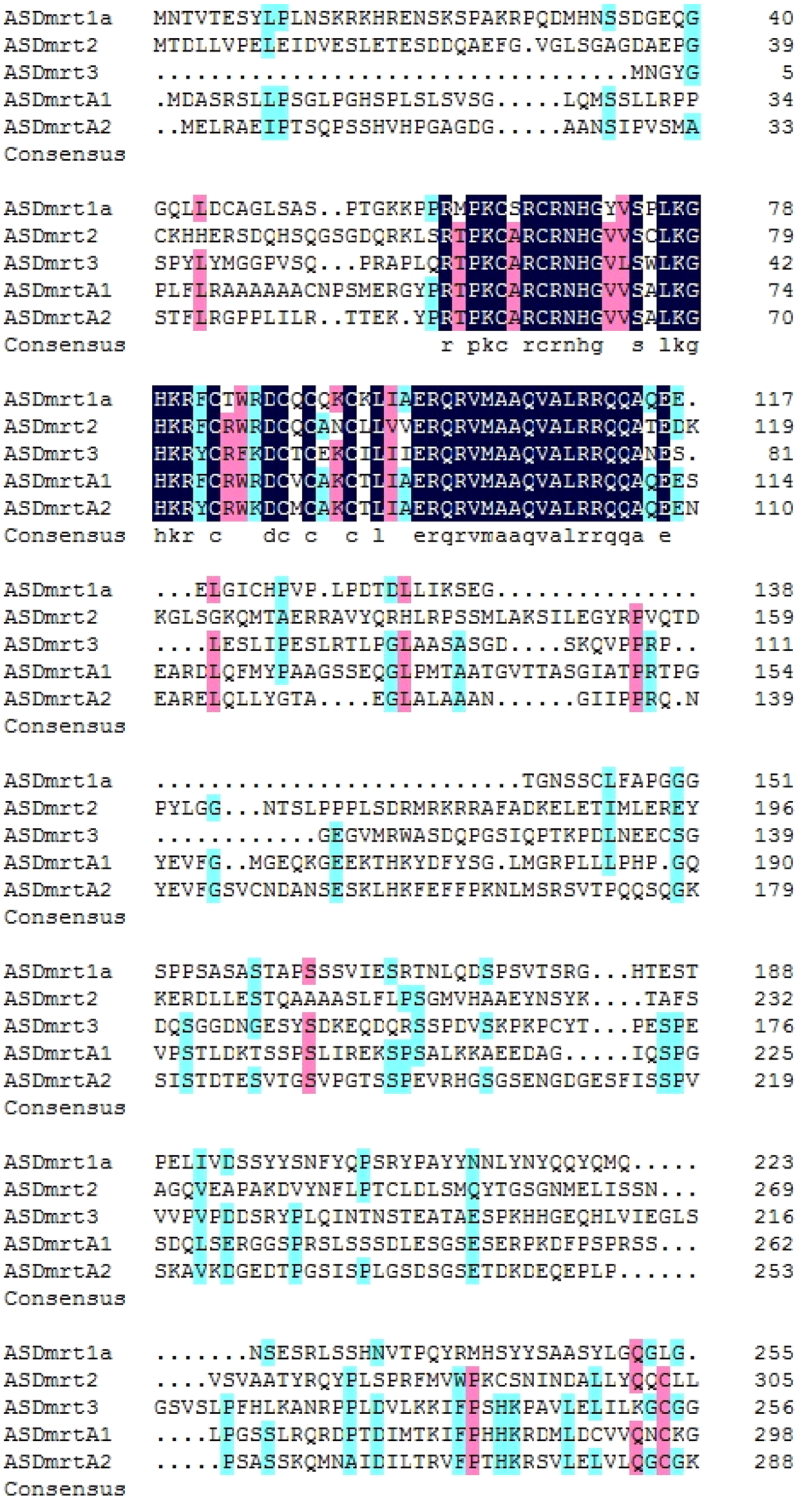

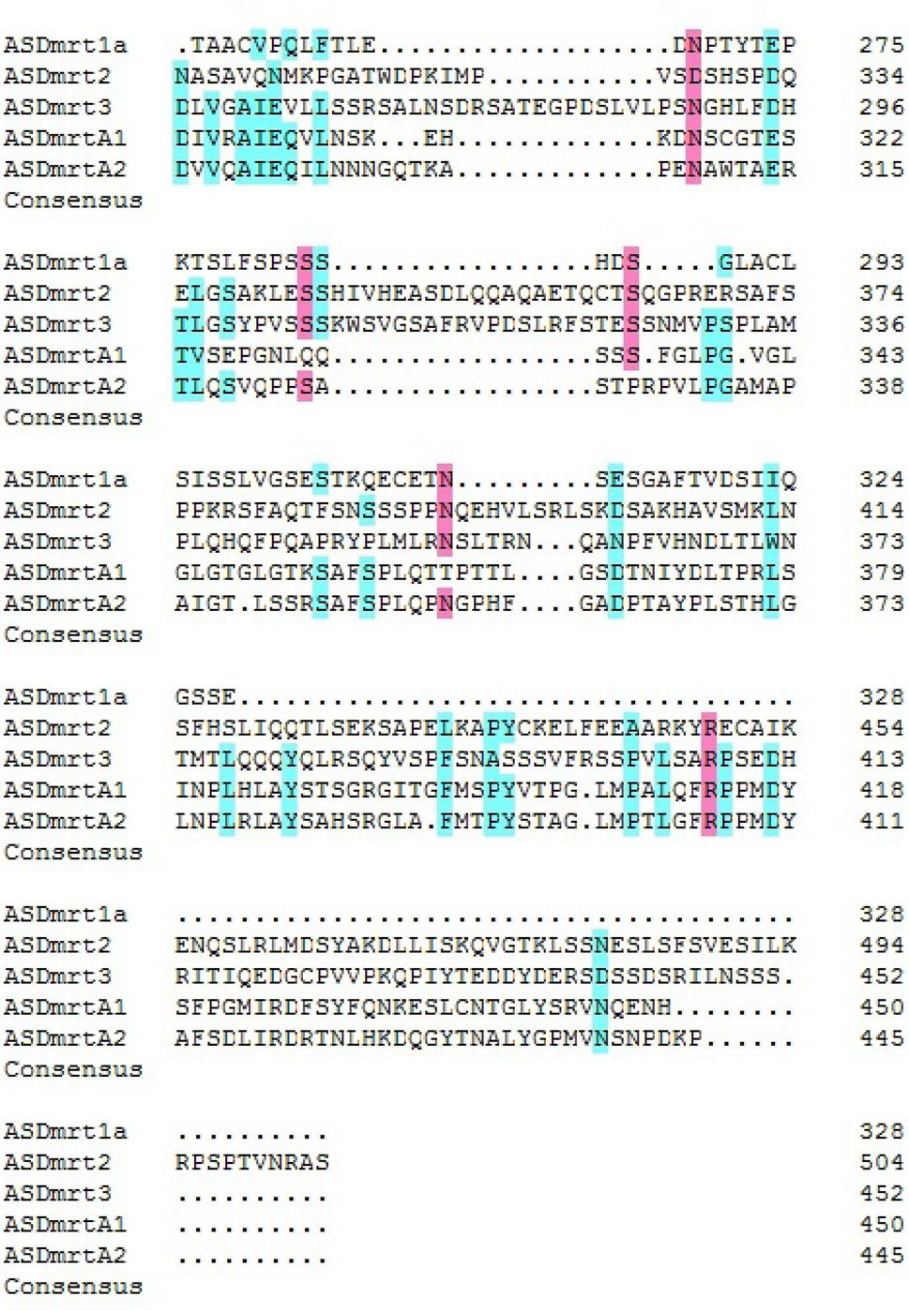
(**A**) Multiple sequences alignment of Dmrt amino acid sequences of Chinese sturgeon. (**B**) Multiple sequences alignment of Dmrt amino acid sequences of Chinese sturgeon.

**Figure 2:**
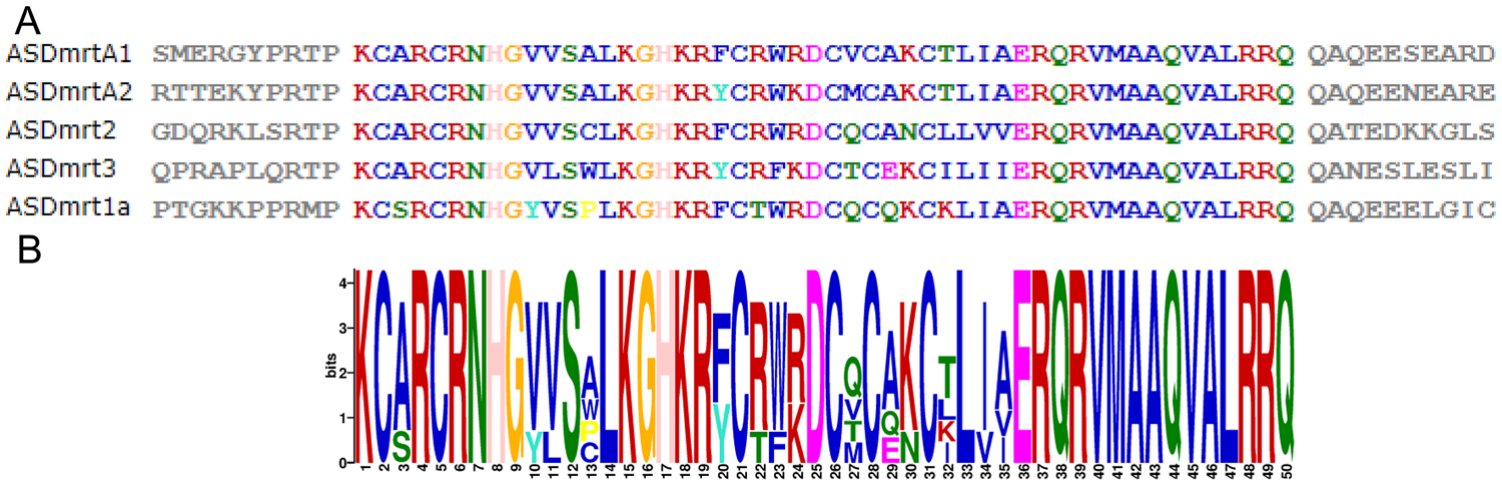
Multiple alignment of domain sequences of Chinese sturgeon Dmrt proteins. (**A**) Mega 4.0 software was used to carry out a multiple alignment of amino acid sequences of the domain of all Chinese sturgeon Dmrt proteins. (**B**) Logo plots of the domain sequence of Chinese sturgeon Dmrt proteins.

Multiple sequences alignments of all predicted Chinese sturgeon Dmrt genes protein sequences were performed using the MEGA4 software (Fig. 1A,B). Sequence alignment showed that the sequences in the Dmrt domain were highly conserved. A total of 54 ASDmrt amino acids were strongly conserved in the protein sequences (Fig. 2).

**Figure 3:**
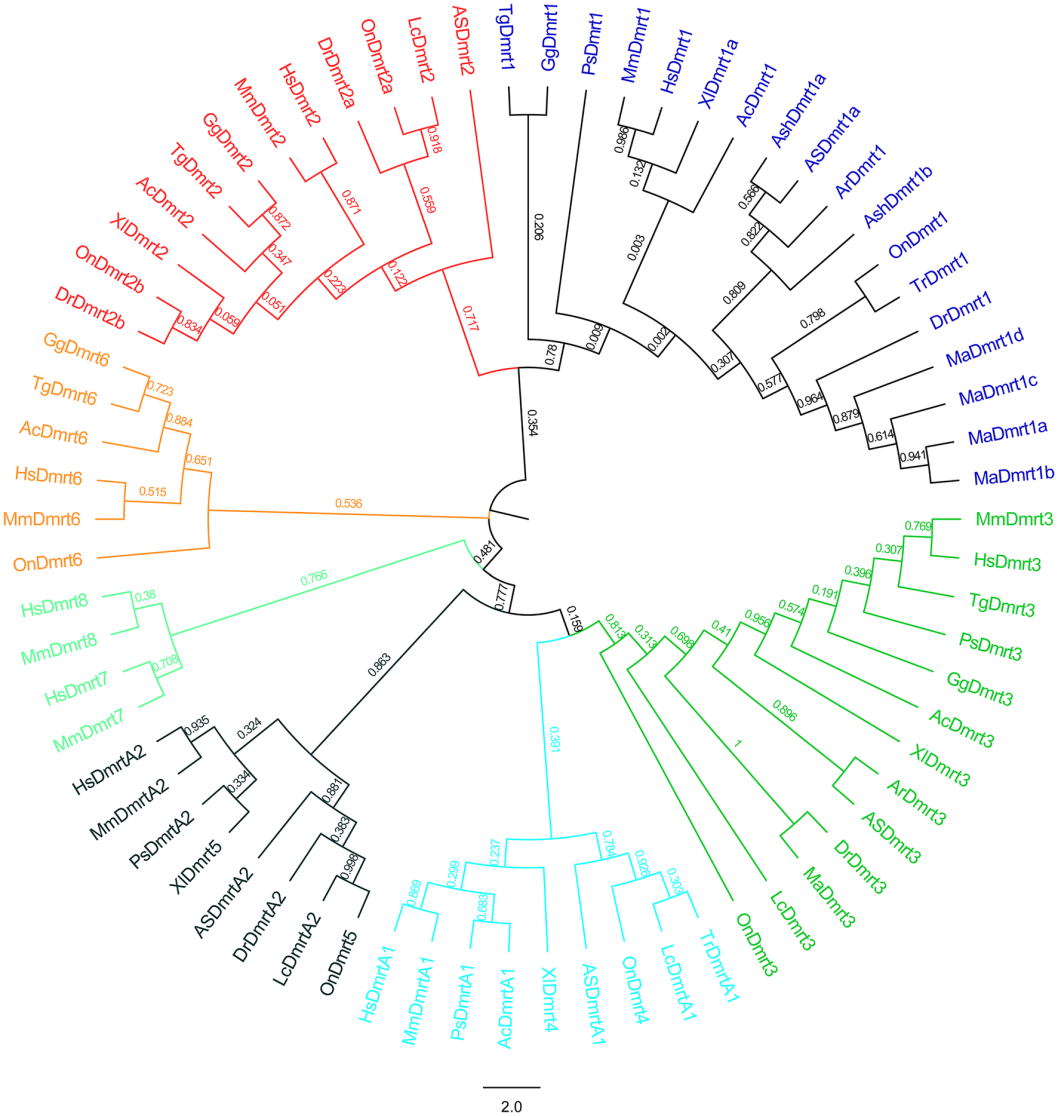
Unrooted phylogenetic tree of Dmrt family proteins (represented by the Dmrt genes from Chinese sturgeon, Zebrafish, Nile Tilapia, African clawed frog, Green Anole, zebra finch, Chicken, Mouse, Human, Amur sturgeon, Sterlet, tiger puffer, Bluntsnout bream, Chinese softshell and Asian seabass).

To analyze the evolutionary relationships of Dmrt genes in Chinese sturgeon, Zebrafish, Nile Tilapia, African clawed frog, Green Anole, zebra finch, Chicken, Mouse, Human, Amur sturgeon, Sterlet, tiger puffer, Bluntsnout bream, Chinese softshell and Asian seabass, an unrooted phylogenetic tree was constructed using full-length amino acid sequences (Fig. 3). Based on phylogenetic analysis, the Dmrt genes of vertebrates can be divided into 8 clades, including Dmrt1, Dmrt2, Dmrt3, Dmrt4, Dmrt5, Dmrt6, Dmrt7 and Dmrt8. As expected, there was no Chinese sturgeon Dmrt genes cluster in the mammalian Dmrt groups (Dmrt7 and Dmrt8).

### Structural analysis and motif composition of Chinese sturgeon Dmrt genes

**Figure 4:**
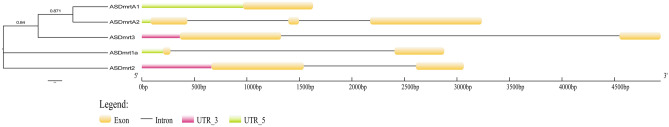
Phylogenetic relationship and the exon–intron structures of the Chinese sturgeon Dmrt family genes.

Gene structure analysis plays important role in the studying the gene function, organization and evolution. To characterize the structural diversity of Chinese sturgeon Dmrt genes, the full-length cDNA sequences and their corresponding genomic DNA sequences were compared. The exon–intron organizations of all the identified ASDmrt genes were examined to gain more insight into the evolution of the Dmrt family in Chinese sturgeon with the web-based bioinformatics tool GSDS (Fig. 4). As shown in Fig. 4, all ASDmrt genes possessed one to three exons and zero to two introns. ASDmrt1a have one introns and two exons. ASDmrt2 have one introns and two exons. ASDmrt3 have one introns and two exons. ASDmrtA1 have one exons. ASDmrtA2 have three exons and two introns.

**Figure 5:**
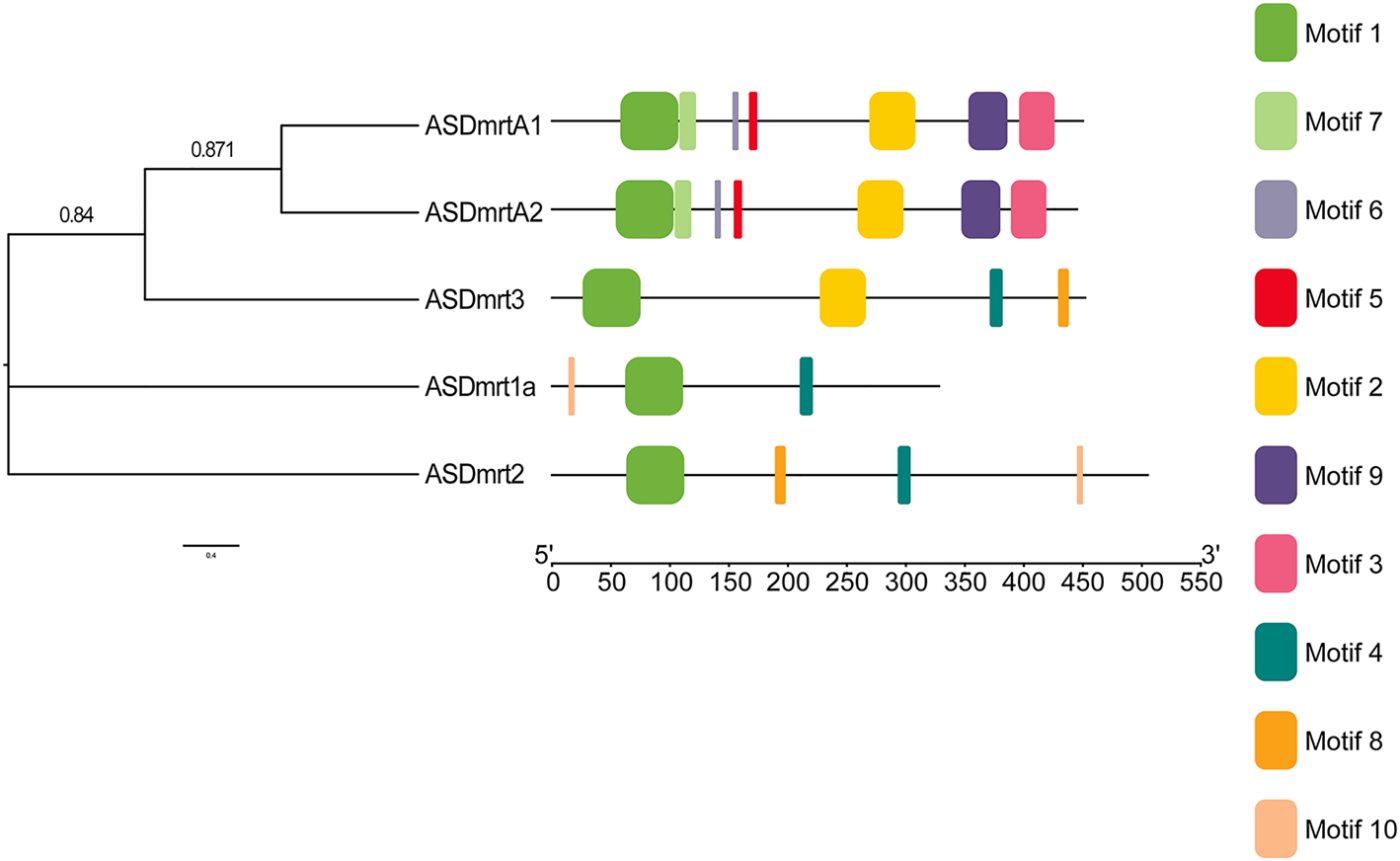
Phylogenetic relationship and the motif composition of Chinese sturgeon Dmrt proteins.

A schematic representing the structure of all ASDmrt proteins was constructed from the MEME motif analysis results (Fig. 5). We found that the motif structures of ASDmrt genes were relatively conserved within the same phylogenetic group, for example, ASDmrtA1 and ASDmrtA2 within the same group generally contained similar motif distribution. Our analysis also showed that ASDmrt members were usually found to share a similar motif composition, other than motif 1 which is the ASDmrt domains widely distributed. For example, ASDmrtA1 and ASDmrtA2 have similar motif distribution, ASDmrt1A, ASDmrt2 and ASDmrt3 all have motif 4, ASDmrt2 and ASDmrt3 both have motif 8, ASDmrt1A and ASDmrt2 both have motif 10. According to domain composition, the five Dmrt family genes could be divided into two groups: the first group contained Dmrt1A, Dmrt2, and Dmrt3, while the second group contained DmrtA1 and DmrtA2. The amino acid conservation schematics of the five DMRT family genes showed that their DM domain sequences were highly conserved.

### The expression profile of ASDmrt genes in hypothalamus, pituitary and gonad of male and female Chinese sturgeon

**Figure 6:**
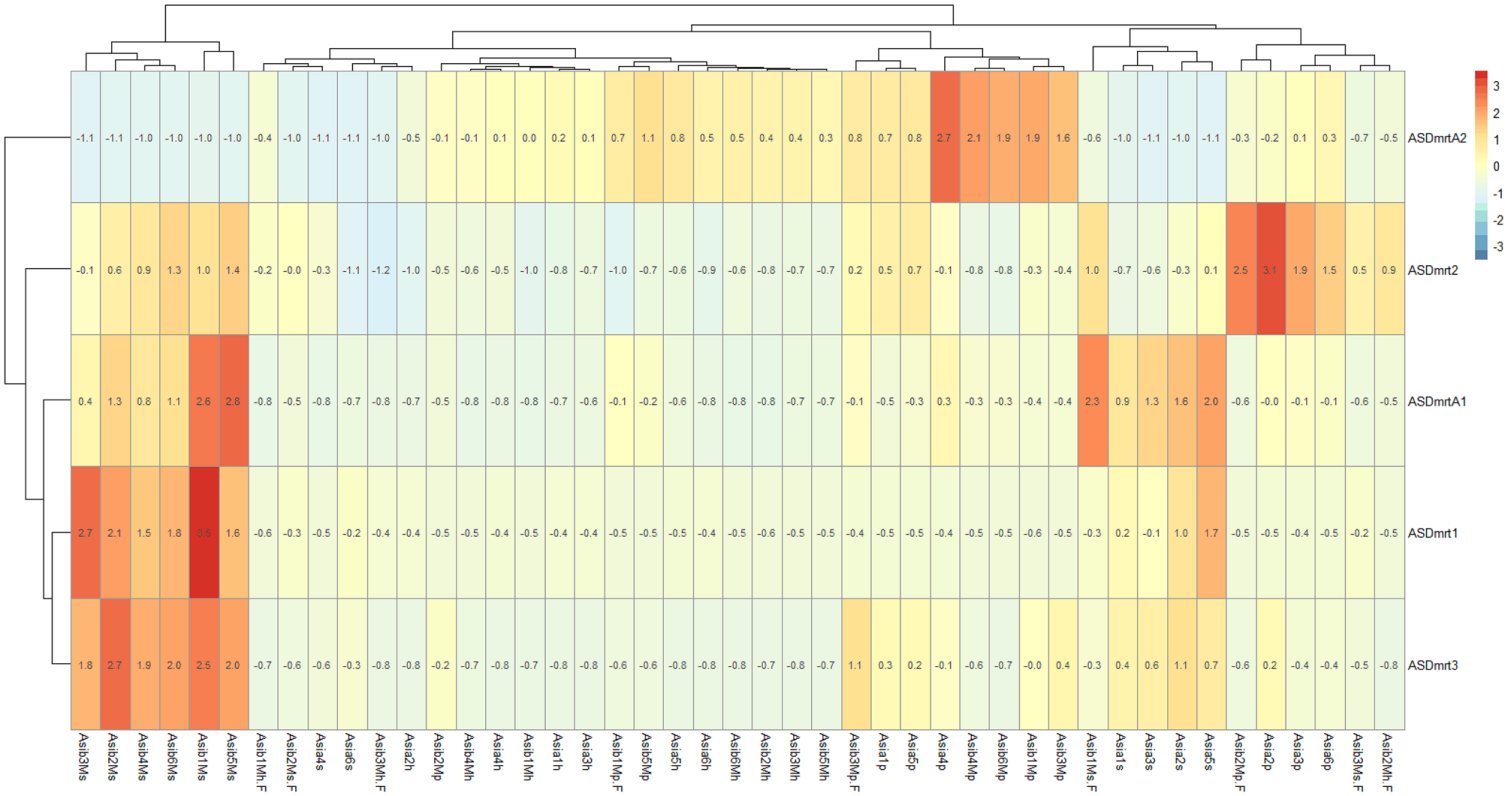
Expression profiles of the Chinese sturgeon Dmrt genes (Asi: Chinese sturgeon; a: gonads entered stage I; b: gonads entered stage II; *M* male, *F* female, *h* hypothalamus, *p* pituitary, *s* gonad; 1–6: the individuals of Chinese sturgeon). Sex is distinguished using gonadal histological analysis.

The expression patterns of all 5 ASDmrt genes in the transcriptome data, which was derived from different developmental stages of Chinese sturgeon tissues, were investigated in this study (Fig. 6). Our results show that the accumulation of Dmrt genes was associated with hypothalamus, pituitary and gonad, and that expression patterns differed between Dmrt genes. ASDmrt1A and ASDmrt3 were highly expressed in the gonad of male Chinese sturgeon. ASDmrt2 was highly expressed in the pituitary of some individuals of Chinese sturgeon. ASDmrtA1 was highly expressed in the gonad of some individuals of Chinese sturgeon. ASDmrtA2 was highly expressed in the pituitary of some individuals of Chinese sturgeon.

**Figure 7:**
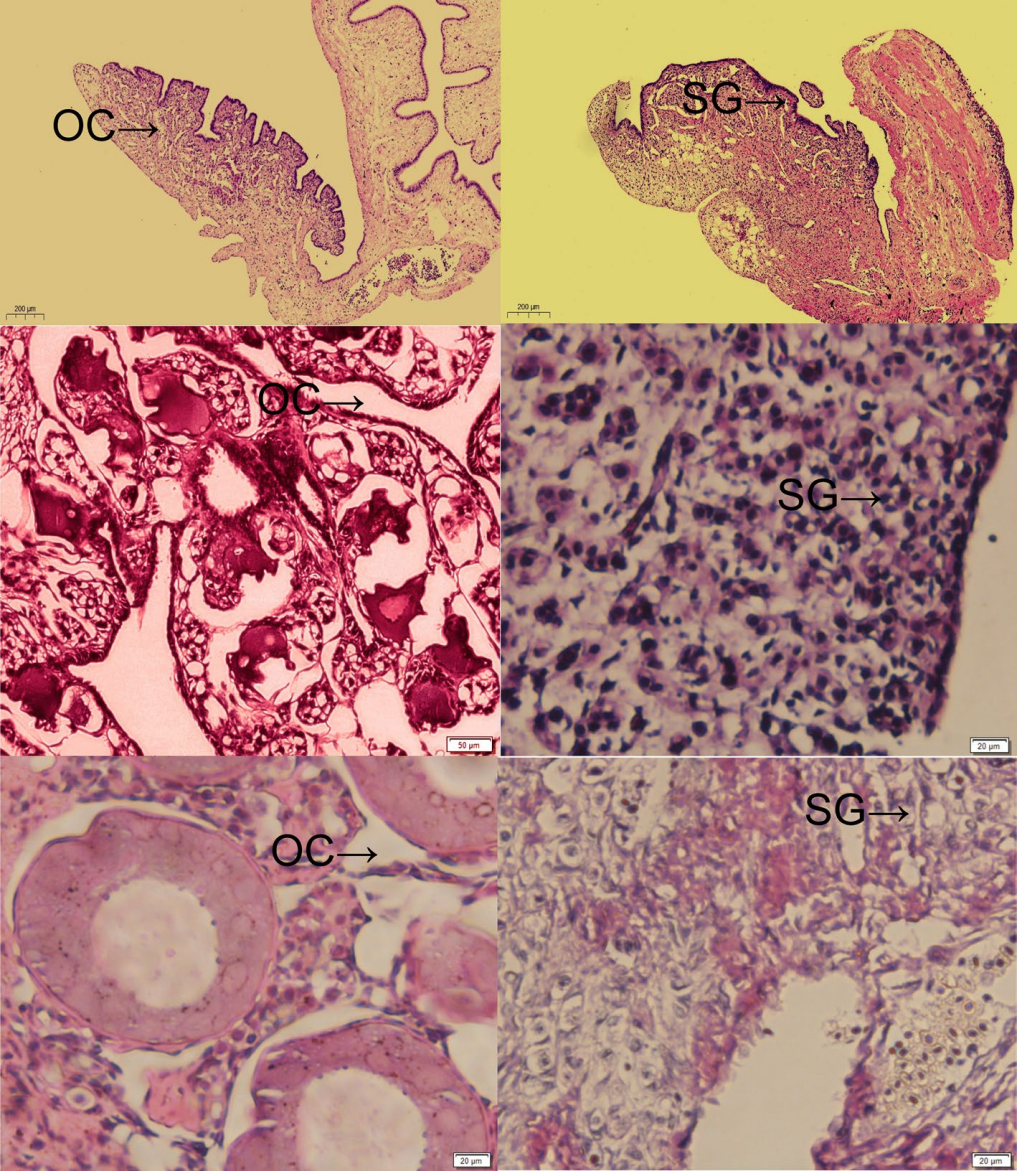
Section of the gonads of Chinese sturgeon (**A**) 1-year-old female Chinese sturgeon; (**B**) 1-year-old male Chinese sturgeon; (**C**) 2-year-old female Chinese sturgeon; (**D**) 2-year-old male Chinese sturgeon; (**E**) 4-year-old female Chinese sturgeon; (**F**) 4-year-old male Chinese sturgeon. *OC* ovary cavity, *SG* spermatogonia.

The gonads of 1, 2, 4-year-old female and male Chinese sturgeon were taken for gonadal histological analysis. Histological section of gonad was showed in Fig. 7. The gonads of 1-year-old Chinese sturgeon are transparent bands in appearance. The hoof tissue and microvessels in the gonads are abundant, and the ovarian margins are wavy folds, but gender could not be distinguished by gonad sectioning in 1-year-old Chinese sturgeon (A and B in Fig. 7). The gonads of 2 year old Chinese sturgeon are white banded. The oocyte has a meridian of about 50–60 µm and may have developed into a primary oocyte. The oocyte is round or polygonal (C and D in Fig. 7). The 4-year old Chinese sturgeon ovary is rich in folds and lobed, the meridian of oocyte is about 150 µm, the oocyte distribution is relatively dispersed (E and F in Fig. 7).

**Figure 8:**
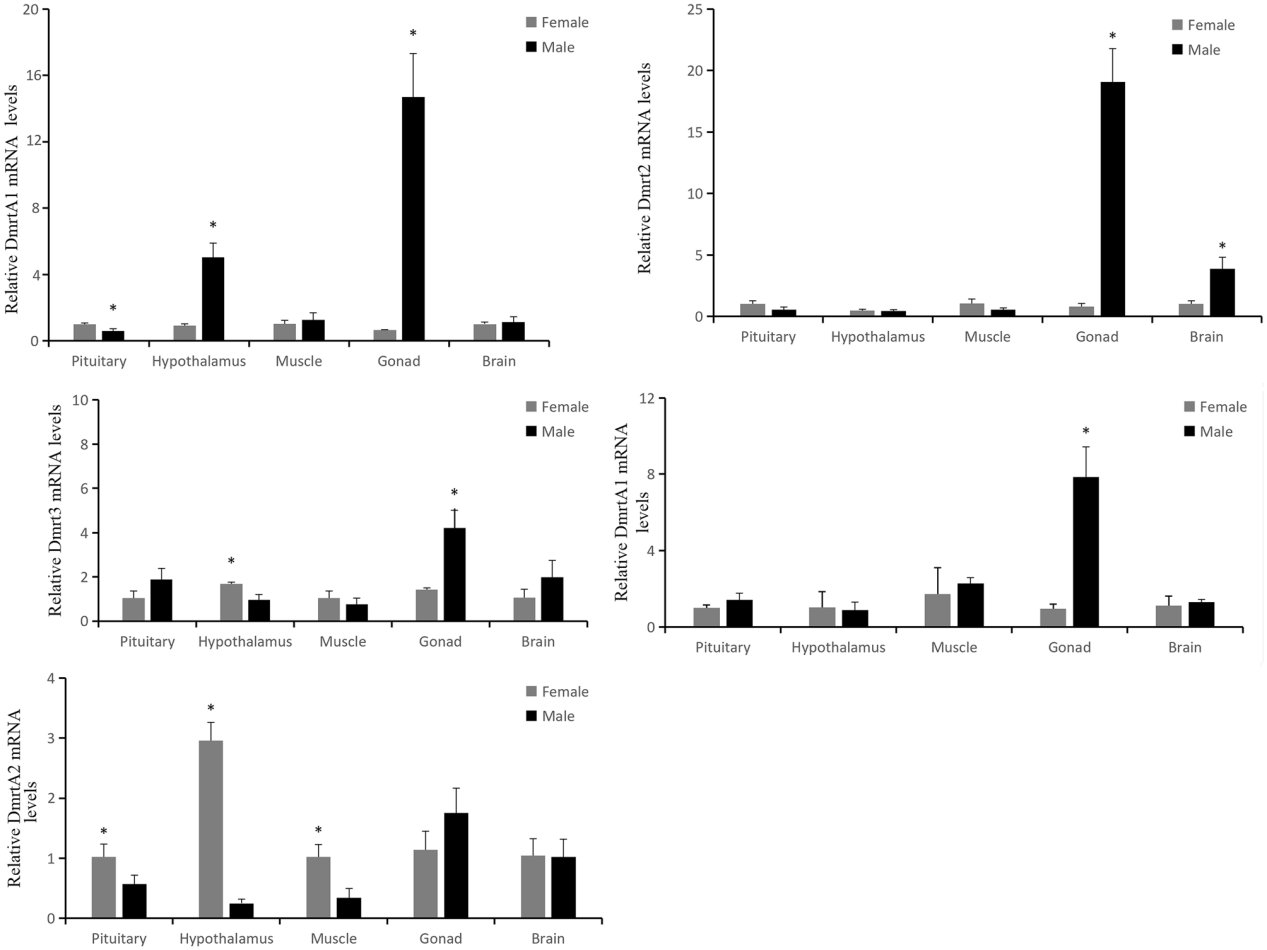
Tissue expression patterns of the five Dmrt family genes in five tissues of 2-year-old Chinese sturgeon individuals. Asterisks (*) above a column represent significant differences between the testis and ovary. Sex is distinguished using the PCR-based sex verification method.

The expression analysis of five Dmrt family genes of Chinese sturgeon was first performed using qPCR. The expression profiles of five tissues (pituitary, hypothalamus, muscle, gonad, and brain) of 2-year-old Chinese sturgeon on the five Dmrt family genes are shown in Fig. 8. This result showed the most highly expressed of ASDmrt1A, ASDmrt2, ASDmrt3, and ASDmrtA1 were in testicle. There was no difference in the expression of ASDmrtA2 between the ovary and the testis.

## Discussion

Dmrt genes have been reported in many fish, such as *Gadus morhua*^30^, *Oreochromis niloticu*^31^, *Takifugu rubripes*^32^. However, genome-wide identification and annotation of Dmrt genes have not been reported in Chinese sturgeon. Our analysis has shown that Chinese sturgeon contain at least 5 Dmrt genes. The number of Chinese sturgeon Dmrt genes is similar of that of African clawed frog and Green Anole, but is higher than that if Zebrafish and zebra finch. The mammalia Dmrt genes (Dmrt7 and Dmrt8) were not present in Chinses sturgeon.

The conserved structural domains of Chinese sturgeon Dmrt genes were assessed in this sturdy. Multiple sequence alignments revealed that the five ASDmrt proteins all have conserved domain with a total of 54 amino acids. The domain gain and loss is a divergent force for expansion of the Dmrt gene family. There was no domain loss event in Chinese sturgeon Dmrt gene family, suggesting ASDmrts were well-conserved.

Comparison of the number of Dmrt genes in Chinese sturgeon with other sequenced vertebrate genomes has shown that Chinese sturgeon possesses comparatively similar number of genes^33^. This also proved that the ASDmrt family is well-conserved in the whole-genome duplication events during the vertebrate evolution.

Numerous studies have proved that Dmrt genes are involved in diverse physiological processes in vertebrate animals, such as neural development, sex determination, and differentiation, organogenesis. Recent findings indicate that members of this gene family are also expressed in other tissues besides the gonads suggesting they may control a broader range of developmental processes. In view of the key roles of Chinese sturgeon Dmrt genes in physiological processes, the expression patterns of ASDmrt genes were investigated in this study basing on the available transcriptome data. By combining gene expression and phylogenetic, new clues to the biological function of Chinese sturgeon Dmrt genes could be inferred through comparison with those function-known Dmrt genes from model vertebrate animals. It would be interesting to functionally characterize those genes in Chinese sturgeon, according to their expression pattern in different tissues. Most Chinese sturgeon Dmrt genes showed higher expression levels in specific tissues (gonad), which could indicate the functional conservation of Dmrt gene family. Some Dmrt genes were more abundant in diverse tissues, indicating their functional differences.

Dmrt1 play an important role in the development of testis. The loss of Dmrt1 can turn the male to female^34,35^. Dmrt1 was only expression in the testis, in comparison with the ovary of the *Oncorhynchus mykiss*^36^. Dmrt1 was highly expression in the testis in comparison with ovary in zebra fish^37^. Some DMRT genes are well-studied in sexual determination and differentiation in somatic cells of the gonads. DMRT1 is a regulator of testicular formation and/or male determination in gonadal somatic cells in various vertebrate species. In chickens, the Z-linked dmrt1 induces male sex determination by its gene dosage^13^. The dmrt1 paralogs, the Y-linked dmy/dmrt1by in teleost fish (*Oryziaslatipes*) and the W-linked dmw in the African clawed frog (*Xenopus laevis*) are sex-determining genes^38–40^. Our result was consistent with previous studies. ASDmrt1A was mainly expression in the testis, except one individual which was phase I of gonads. Therefore, we can speculate this individual was male Chinese sturgeon.

Dmrt2 palys an important role in the differentiation of somite in mouse^41^. In the zebrafish, Terra (Dmrt2) is transiently expressed in the presomitic mesoderm and in the newly formed somites. It is one of the first reported Dmrt genes expressed outside the gonads^42^. In this organism overexpression of Terra induces apoptosis in the somitic mesoderm, suggesting that Terra expression levels need to be tightly regulated for proper mesoderm development. Dmrt2a plays an important role in the development of somite in zebrafish while Dmrt2b^43^. ASDmrt2 was mainly expressed in the pituitary in comparison with the gonad. This may prove that ASDmrt2 had little effect on the development of gonad. ASDmrt2 may play role in the earlier stage of gonad development.

Dmrt3 was took part in the development of gonad in mouse^44^. Our analysis was shown that ASDmrt3 was expressed highly in the testis. This may prove ASDmrt3 also took part in the development of gonad in Chinese sturgeon.

Dmrt4 null mice develop essentially normally, undergo full sexual differentiation in both sexes and are completely fertile, indicating that Dmrt4 is not required for the development of the mouse gonads^45^. However, two phenotypes have been described in Dmrt4 mouse mutants. First, the ovaries of most mutant females present polyovular follicles, suggesting that Dmrt4 regulates folliculogenesis, a process during which oocytes are incorporated into primordial follicles. Second, mutant males exhibited mounting behavior toward other males. Dmrt4 was expressed in the testis and ovary in the Japanese pufferfish^46^. Dmrt4 was only expressed in the gonad, pituitary and hypothalamus in *Oreochromis aureus*^47^. ASDmrtA1 was highly expressed in the testis and ovary in comparison with pituitary and hypothalamus. ASDmrtA1 was important for development of gonad for male and female Chinese sturgeon.

Dmrt5 was not expressed in the gonad in the Japanese pufferfish while it was expressed in the spleen^46^. This may prove the Dmrt5 took part in the formation of the immune system. Dmrt5 was highly expressed in the brain in comparison with gonad, kidney, lungs, stomach and heart in the mouse^44^. ASDmrtA2 was highly expressed in the pituitary in comparison with the gonad and hypothalamus. Notably, the new data revealing high expression of dmrt1A in the hypothalamus and testis of 2-year-old males, with dmrtA2 expressing in the female hypothalamus (Fig. 8), adds an intriguing dimension to the understanding of the Dmrt gene family’s role in central nervous system and sexuality.

These divergences in the expression of Dmrt genes across species indicate that the expression patterns and presumably the function of some members of this gene family may have shifted during evolution.

## Conclusion

We have identified and characterized Chinese sturgeon Dmrt gene family for the first time. A total of 5 putative Dmrt genes were identified. The gene structure, conserved protein domain and the phylogenetic relationship of Dmrt gene family were systematically analyzed. The expressed profile of Chinese sturgeon Dmrt genes in gonad, pituitary and hypothalamus in the male and female were investigated. The results indicated that the accumulation of Dmrt genes was involved in different tissues, and the expression profile also differed among each Dmrt genes. ASDmrt1A, ASDmrt2, ASDmrt3, and ASDmrtA1 showed testis-dominant expression in Chinese sturgeon in the present study. Therefore, those genes played an important role in the development of testicle, and may be useful tool in distinguishing between male and female of Chinese sturgeon, which is worth further study. Our study represents a comprehensive overview of the Dmrt gene family in Chinese sturgeon and provides new insights into the evolution of this gene family, increases available knowledge for the further investigation of Dmrt functions in sex differentiation in sturgeon.

## Data Availability

The datasets generated and/or analysed during the current study are available in the Mendeley Data repository, 10.17632/db7rks2ybw.1 (https://data.mendeley.com/datasets/db7rks2ybw/1).
